# Advanced detection of coronary artery disease via deep learning analysis of plasma cytokine data

**DOI:** 10.3389/fcvm.2024.1365481

**Published:** 2024-03-08

**Authors:** Muhammad Shoaib, Ahmad Junaid, Ghassan Husnain, Mansoor Qadir, Yazeed Yasin Ghadi, S. S. Askar, Mohamed Abouhawwash

**Affiliations:** ^1^ Department of Computer Science, CECOS University of IT and Emerging Sciences, Peshawar, Pakistan; ^2^ Department of Computer Science, Al Ain University, Al Ain, UAE; ^3^ Department of Statistics and Operations Research, College of Science, King Saud University, Riyadh, Saudi Arabia; ^4^ Department of Computational Mathematics, Science and Engineering (CMSE), College of Engineering, Michigan State University, East Lansing, MI, United States; ^5^ Department of Mathematics, Faculty of Science, Mansoura University, Mansoura, Egypt

**Keywords:** coronary artery disease, convolutional neural network, recurrent neural network, supervised learning, plasma cytokines

## Abstract

The 2017 World Health Organization Fact Sheet highlights that coronary artery disease is the leading cause of death globally, responsible for approximately 30% of all deaths. In this context, machine learning (ML) technology is crucial in identifying coronary artery disease, thereby saving lives. ML algorithms can potentially analyze complex patterns and correlations within medical data, enabling early detection and accurate diagnosis of CAD. By leveraging ML technology, healthcare professionals can make informed decisions and implement timely interventions, ultimately leading to improved outcomes and potentially reducing the mortality rate associated with coronary artery disease. Machine learning algorithms create non-invasive, quick, accurate, and economical diagnoses. As a result, machine learning algorithms can be employed to supplement existing approaches or as a forerunner to them. This study shows how to use the CNN classifier and RNN based on the LSTM classifier in deep learning to attain targeted “risk” CAD categorization utilizing an evolving set of 450 cytokine biomarkers that could be used as suggestive solid predictive variables for treatment. The two used classifiers are based on these “45” different cytokine prediction characteristics. The best Area Under the Receiver Operating Characteristic curve (AUROC) score achieved is (0.98) for a confidence interval (CI) of 95; the classifier RNN-LSTM used “450” cytokine biomarkers had a great (AUROC) score of 0.99 with a confidence interval of 0.95 the percentage 95, the CNN model containing cytokines received the second best AUROC score (0.92). The RNN-LSTM classifier considerably beats the CNN classifier regarding AUROC scores, as evidenced by a *p*-value smaller than 7.48 obtained via an independent *t*-test. As large-scale initiatives to achieve early, rapid, reliable, inexpensive, and accessible individual identification of CAD risk gain traction, robust machine learning algorithms can now augment older methods such as angiography. Incorporating 65 new sensitive cytokine biomarkers can increase early detection even more. Investigating the novel involvement of cytokines in CAD could lead to better risk detection, disease mechanism discovery, and new therapy options.

## 1 Introduction

The crucial role inflammation plays in the beginning and development of coronary heart disease is well-known (CHD) (1). However, the precise mechanism through which inflammation contributes to the pathophysiology of CHD remains unclear (2, 3). Several inflammatory indicators have been evaluated for their capacity to predict CHD risk, with C-reactive protein (CRP) receiving the most attention. On the other hand, CRP does not appear to be a risk factor for coronary heart disease. Cytokines are chemical messengers produced by immune and nonimmune cells that influence cell activity. The liver also produces C-reactive protein (CRP), an inflammation marker and cardiovascular disease risk factor (4). Cytokines are chemical messengers produced by the immune system and other cells in order to regulate numerous biological processes. Activated macrophages, T cells, B cells, and other immune and nonimmune cells generate them. They are crucial in immune response modulation, tissue repair, and homeostasis maintenance. Understanding their biology is essential for developing new treatments for chronic inflammatory diseases such as autoimmune disorders and cancer. Apart from their role in the development of well-known CHD risk factors such as tobacco use and high cholesterol, hypertension (blood pressure), and diabetes, these factors also initiate a cascade reaction in which the release of very low-concentration cytokines recruits inflammatory cells, which then produces additional cytokines, amplifying the local inflammatory response. (TNF-α) and interferon-γ (IFN-γ) are proinflammatory cytokines that play an essential role in developing chronic inflammatory illnesses such as coronary artery disease (CAD) (5). These cytokines are present in the circulation and drive monocytes and macrophages to create interleukin-1 (IL-1) and interleukin-6 (IL-6), influencing the endothelial cells lining the artery wall.

Interleukin-6 (IL-6) is an important proinflammatory cytokine genetically linked to coronary artery disease. It has been established that elevated levels of IL-6 are related to an increased risk of coronary heart disease, and it is believed to play a crucial role in the onset and progression of the inflammatory process that leads to coronary plaque formation (6). It is well-recognized that IL-6 stimulates the production of other cytokines, chemokines, and proinflammatory chemicals. It also encourages the development of smooth muscle cells in the artery wall, contributing to plaque formation. In addition, IL-6 is known to play a role in developing other inflammatory diseases, such as rheumatoid arthritis and some kinds of cancer, emphasizing the necessity of knowing its involvement in the pathogenesis of chronic inflammatory disorders (7). CANTOS provides crucial insight into the link between inflammation and coronary artery disease. Chronic inflammation is connected with an increased risk of coronary heart disease (CHD). The findings indicate that targeting IL-1 with anti-IL-1 medications such as canakinumab can considerably lower the risk of severe adverse cardiovascular events in those with elevated levels of the inflammatory biomarker C-reactive protein (CRP) (8, 9). Participants had a prior myocardial infarction (MI) and CRP levels greater than 2 g/L. As part of the study, they were given canakinumab, which decreases interleukin-6 levels in the bloodstream. Scientists are now investigating the effects of other cytokines on the risk of coronary heart disease, considering the findings of Mendelian randomization studies (MR studies) and the CANTOS trial.

In Europe and North America, cardiovascular disease is the leading cause of mortality (10), emphasizing the importance of incorporating developing risk variables to enhance risk prediction, enable early diagnosis, and customize care. The ability of machine learning like CNN (11) and LSTM is used to achieve patterns that can be used to inform healthcare decisions (12). By adjusting various tuning settings and combining k-fold cross-validation, we demonstrate the juxtaposition of algorithm CNN and LSTM in this study. This effective resampling strategy eliminates the problem of overfitting and improves model generalization (13). Due to data availability constraints, the Random Oversampling Example (ROSE) Technique improves and balances the data before model construction and final prediction. ROSE, accessible via the Integrated R Archive ‘Network = (https://cran.r.project.org/web/Packages/ROSE/index.html), uses a smooth bootstrap strategy to simulate balanced synthetic data.

To improve the classification of individuals with or without clinical coronary artery disease, we have used 450 plasma cytokines as novel biomarkers (CAD). This method has the potential to uncover disease pathways involving previously unknown cytokine targets and enhance early detection of people at risk. In response to cellular signals, the immune system produces cytokines, which are proteins. By targeting active receptors and generating downstream signals, they operate as messengers to other cells. Lymphokines, chemokines, interferons, and interleukins are examples of cytokines responding to environmental signals that initiate an anti-inflammatory or pro-cascade response (14, 15). Cytokines have been linked to the progression and development of coronary artery disease (9).

The study included 1,040 people to create a dataset with various biomarker levels. Every one of the 450 cytokine indicators was put to the test. Based on the final target attributes, people were assigned to the CAD (421 individuals) or control groups (619 individuals). The 36 cytokine biomarkers were merged in the model's feature space to quantify similarity. Deep machine learning techniques such as CNN and LSTM are used to create patterns that can be used to guide healthcare decisions. The CNN and RNN-LSTM are commonly used when developing a computer-aided diagnosis system. The primary contribution of this research work is the creation of standard data labeled by specialists in the relevant field. Using the deep neural network designer application found in MATLAB, a custom CNN, and LSTM network architecture is developed for the detection of coronary artery disease. Both the architecture and the parameters of the networks were designed following the dataset's characteristics.

This research study makes significant contributions in several key areas that are discussed as follows:

• The study introduces an innovative approach to the detection of CAD by leveraging deep learning techniques, specifically CNN and RNN-LSTM, for the analysis of plasma cytokine data.
• The integration of deep learning algorithms, CNN and RNN-LSTM, with a comprehensive set of 450 plasma cytokine biomarkers represents a novel and advanced methodology. This approach provides a more nuanced understanding of the complex interplay between cytokines and CAD, surpassing traditional methods.
• The study achieves high diagnostic accuracy, as evidenced by Area Under the Receiver Operating Characteristic curve (AUROC) scores. The RNN-LSTM classifier, in particular, outperforms the CNN classifier, showcasing the potential of deep learning in improving the accuracy of CAD detection using plasma cytokine data.
• By employing deep learning techniques, the study identifies key cytokine biomarkers associated with CAD risk. This not only contributes to the understanding of the disease mechanisms but also provides potential targets for future therapeutic interventions.
• The study highlights the application of machine learning, specifically CNN and RNN-LSTM, in enhancing cardiovascular risk prediction. The models developed showcase the potential of these algorithms as valuable tools for healthcare professionals in making informed decisions regarding CAD diagnosis.
• The study conducts a comprehensive comparison with state-of-the-art (SOTA) approaches, demonstrating the superior accuracy of the proposed deep learning model. This emphasizes the effectiveness of the novel approach in outperforming existing methods commonly employed in CAD detection.
• The identification of specific cytokine biomarkers associated with CAD risk holds the potential for personalized medicine. Tailoring interventions based on individual cytokine profiles may lead to more targeted and effective treatment strategies, contributing to improved patient outcomes.

This article is organized logically and coherently to thoroughly analyze the subject at hand. Section 2’s literature review provides an in-depth analysis of existing research on the topic as a basis for the research. The third section describes the study approach, including a description of the dataset, the suggested model framework, the training of the hyper models, and the processes for optimizing the hyperparameters to produce optimal feature weights. Section 4 describes the performed experiments, performance evaluations, and model performance comparisons. In this section, the outcomes of the study are discussed. In Section 5, the paper's principal results and recommendations for future research are outlined. Overall, the format of the article is intended to provide a complete and clear comprehension of the conducted research and its outcomes.

## 2 Literature review

In literature, high-level methods like RNNs & CNNs are widely anticipated to extract patterns for verifications, judgments, and treatments (16). Authors in (13) illustrate the “exploratory juxtaposition of “CNN” and RNN’ by modifying various tuning settings and combining k-fold cross-validation. This successful resampling method solves the overfitting problem and improves the model's generalization. The Random Oversampling Example Technique (ROSE) enriches and balances the data prior to model creation and final prediction due to data availability constraints (17). The Integrated R Archive Network hosts ROSE, which puts on balanced synthetic data using a smoothing bootstrap technique.

A comprehensive analysis of biomarkers was conducted to determine whether individuals with or without clinical coronary artery disease (CAD) are at risk of developing the condition. Four hundred fifty plasma cytokines were utilized as novel biomarkers in this endeavor. Using multiple biomarkers in combination allowed for the development of a robust and accurate classification system for CAD. This approach represents a significant advancement in identifying and diagnosing this prevalent disease. The utilization of these biomarkers may have the potential to improve the detection and management of CAD. The method can help to uncover disease pathways involving previously undiscovered cytokine targets and improve early diagnosis in high-risk people (18). The immune system creates cytokines, or proteins, reacting to cellular signals. Cytokines such as “lymphokines,” “chemokine,” “interferon,” and interleukins respond to environmental signals to launch a pro- or anti-inflammatory cascade reply (14, 19). The development and progression of coronary artery disease have been linked to cytokines (9).

Hampel et al. (20) proposed using machine learning techniques to investigate cardiac computed tomography (CT) visualization. Due to a scarcity of experienced cardiac imagers and the severe workload of medical practitioners, CT provides detailed high 3-D images with hundreds100 of underutilized slices (21). By producing reliable and fast answers, machine learning algorithms can overcome the limitations of manual diagnosis, potentially leading to further secondary diagnoses. Over the last ten years, machine learning techniques have improved CAD detection and characterization, according to this survey. Despite the obstacles of using ML in a clinical setting, the power of novel ML algorithms drives significant discoveries in CAD classification, according to the findings (22).

Due to the nature of ML algorithms learning from prior estimates, “Martin-Isla, Carlos, et al.” (23). Examined the use of (“ML”) algorithms for image-based CAD diagnosis that has enabled deeper eligibility and more excellent diagnosis. Furthermore, a considerable amount of literature in this field emphasizes the potential of machine learning techniques in CAD identification.

The inflammatory profile of mature patients with the disease of mouth, foot, and hand, joint in the Asia-Pacific area, was studied using cytokines by Ling-Hua Yu et al. (15). Participants used a random forest to identify the (HFMD) illness collection from controls using 26 key cytokines as predictive characteristics (24). The study revealed links between enterovirus infection, genotype, and clinical manifestation. The algorithm RNN-LSTM ended up with an AUROC value of 0.91, exhibiting its partitioning solid ability. Stevens et al. (25) Working cytokine predictors to discriminate the disease malaria from bloodstream bacterial infections using RNN-LSTM, implying that cytokines are active and predictive solid biomarker profiles. The 6–15 cytokines employed for the job were chosen using ML classification algorithms. Researchers employed cytokines to discriminate severe malaria contagion from asymptomatic malaria to compensate for the absence of quick malaria diagnosis. This study found that 88% of disease states could be predicted accurately, which could lead to the development of new rapid diagnostics in Sub-Saharan Africa.

Saini examined the application of CNN for recognizing QRS complex waves in ECG-related data, Indu et al. (26). The authors demonstrated that the k value and the classification distance metric primarily determine prediction accuracy. Experiments showed that the Euclidean distance value and k = 3 are the best when paired with a 6-fold cross-validation-produced CNN classifier. Forecast accuracy reached 99 percent, which is extremely high.

A CANTO (canakinumab anti-inflammatory thrombosis outcomes) was an objective assessment funded by Novartis that aimed to investigate the role of interleukin 1 in inflammation at the cellular level. R, Paul M et al. The males in the study will get monoclonal antibodies against interleukin one beta as part of the trial. Canakinumab, an anti-inflammatory drug that targets the interleukin one beta innate immune pathway every three months the, is given 150 mg. Compared to placebo, there was a considerably decreased incidence of cardiovascular events unrelated to cholesterol lowering in the research. This research aims to improve and extend the approaches employed in previous investigations. The primary goal is to improve separability for AUROC curve assessment by combining sophisticated algorithms, such as the “CNN” and RNN (LSTM), with new cytokine-biomarkers.

Almost half of all deaths worldwide are caused by heart disease or stroke, according to Lozano et al. (27). Both diseases are significant public health issues in developing countries, notably Africa. Significant socioeconomic disparities in heart disease and stroke morbidity and mortality are common in many Western countries, particularly the United States (28). Although there have been improvements in reducing disparities in morbidity and mortality related to heart disease and stroke, the gap between rich and poor is expected to widen over the next several years in several countries. This is particularly evident when considering the persistent disparities between socioeconomically disadvantaged groups and their more affluent counterparts. Implementing practical strategies and preventive interventions is crucial to address the health disparities related to heart disease and stroke, especially among older adults, in the context of socioeconomic inequality. Doing so can effectively reduce health inequalities and promote better overall health outcomes. According to Mattiussi and Lippi (29), approximately 70% of heart disease and stroke cases occur in individuals over the age of 65, indicating that heart disease and stroke are diseases of aging (Yousuf et al.) (30). The World Health Organization reports that as populations age and the income disparity between rich and poor continues to widen, the global incidence of heart disease and stroke continues to rise, particularly in low- and middle-income countries (LMICs) such as China (31). Few studies have examined the relationship between socioeconomic status and heart disease and stroke; evidence linking these diseases to socioeconomic status is still scarce in low- and middle-income countries, particularly developing countries. Most evidence from low- and middle-income countries comes from hospital-based studies involving various middle-aged populations of varying quality (31). According to the International Society for Cardiovascular Diseases (ISCD), the relationship between socioeconomic status (SES) and mortality from heart disease and stroke in older adults in low- and middle-income countries needs to be better understood (LMICs). As a result, it is primarily concerned with China's older adults, the subject of this thesis, which examines their relationship with them.

Numerous studies have been conducted to determine which parameters or factors significantly improve the efficacy of CRT. These studies’ findings were published in the journal Radiology. Park et al. (32) demonstrated that using SPECT images to assess the LV's most recent activation can improve both the speed with which the LV is placed and the quality of the CRT response. Machine learning techniques have been employed in various studies to predict mortality in patients with coronary artery disease and other ailments. For instance, researchers effectively employed machine learning to identify patients with CRT (Cardiac et al.) who would benefit from the phenol group. This approach enabled them to observe the improvements in CRT response over time. The researchers utilized unsupervised learning methods to identify patients more likely to respond positively to the treatment. They combined clinical characteristics with echocardiographic data, precisely measurements of myocardial infarction. They left ventricular volume changes throughout the cardiac cycle to identify patients with a higher likelihood of positive response. According to the findings, it is possible to classify heterogeneous groups of heart failure patients in a clinically meaningful manner using an unsupervised machine learning technique, which can aid in the identification of subgroups of patients who are most likely to respond to specific therapies. According to the paper's authors, a prospective controlled trial should assess the proposed model's feasibility in patients with phenotypic heart failure and clinical decision-making.

The research article outlines (33), a randomized trial examining the impact of low-dose colchicine (0.5 mg daily) vs. a placebo on patients who recently experienced a myocardial infarction. Involving 4,745 participants over 22.6 months, the trial revealed a significant reduction (5.5% vs. 7.1%, hazard ratio 0.77) in the primary composite endpoint, including cardiovascular death, myocardial infarction, and stroke. Noteworthy reductions were observed in death from cardiovascular causes, stroke, and urgent hospitalization for angina leading to coronary revascularization. Diarrhea, the primary adverse event, occurred more frequently in the colchicine group (17% vs. 8.9%). The study concludes that low-dose colchicine effectively reduces cardiovascular events, presenting a potential cost-effective treatment for coronary artery disease.

This study presents a meta-analysis of randomized controlled trials (RCTs) assessing the efficacy and safety of colchicine in post-acute coronary syndrome (ACS) patients (34). Encompassing two RCTs and 5,540 participants (50.1% colchicine, 49.9% placebo), the primary outcome was major adverse cardiovascular events (MACE), with secondary endpoints including stroke, myocardial infarction (MI), all-cause and cardiovascular death, and urgent revascularization. Results indicate a significant reduction in the colchicine group for the composite endpoint (5.5% vs. 7.6%, OR 0.67, 95% CI 0.46–0.98, *p* = 0.04, *I*^2^ = 46%). Notably, colchicine demonstrated substantial reductions in cerebrovascular accidents (OR 0.31, 95% CI 0.14–0.69, *p* = 0.004, *I*^2^ = 0%) and repeat revascularization (OR 0.36, 95% CI 0.14–0.90, *p* = 0.03, *I*^2^ = 54%). Nevertheless, no significant differences were observed in cardiovascular death, non-cardiovascular death, MI at longest follow-up, or resuscitated cardiac arrest between the colchicine and placebo groups.

## 3 Methodology

### 3.1 Custom data Set creation

A total of 1,040 people were included in the study, each with different biomarker levels. Each of the 450 cytokine indicators was tested. They assigned People to the CAD (390 individuals) or control groups based on the final target trait qualities (650 individuals). The 36 cytokine biomarkers were merged in the model's feature space to quantify similarity, followed by the CAD or Regulator categorization. They approved this study via the institutional review board human research group & followed the standards of the Declaration of Kuwait Teaching Hospital Peshawar. Prior to participating, all individuals signed a written informed consent form. Blood samples were taken from males (43.3%) and females (43.3%) whose ages ranged from 17 to 64 years (median age = 40). This group included individuals who had been diagnosed with CAD as well as age- and sex-matched controls. The infarction Myocardial, angiographic ally confirmed (CAD), or coronary artery bypass graft surgery was all present in the CAD participants. There was no history of CAD or clinical indication of it in the control group. In our study, we examined the relationship between cytokines and coronary artery disease (CAD), focusing on the role of these signaling proteins in cardiovascular health. We utilized a large dataset of 450 cytokines, each playing a crucial role in immune responses and inflammation. The selected cytokines in Table 1 provide a comprehensive overview of the key players associated with CAD. While the table includes only 10 cytokines, it serves as a representative sample of our extensive analysis.

**Table 1:** Selected cytokines and their role in CAD.

Cytokine	Role in CAD
Tumor necrosis factor-α (TNF-α)	Proinflammatory cytokine, implicated in chronic inflammatory diseases, including CAD.
Interferon-γ (IFN-γ)	Proinflammatory cytokine associated with the development of chronic inflammatory illnesses such as CAD.
Interleukin-1 (IL-1)	Influences endothelial cells lining the artery wall, contributing to CAD development.
Interleukin-6 (IL-6)	Important proinflammatory cytokine genetically linked to CAD; elevated levels are associated with CAD.
Interleukin-10 (IL-10)	Anti-inflammatory cytokine, may have a protective role in CAD by modulating immune responses.
Interleukin-8 (IL-8)	Chemokine involved in recruiting immune cells to inflammation sites; associated with CAD progression.
Interleukin-12 (IL-12)	Proinflammatory cytokine linked to atherosclerosis, contributing to CAD development.
Transforming growth factor-β (TGF-β)	Plays a role in tissue repair but may contribute to fibrosis in CAD; its dysregulation is linked to CAD.
Monocyte chemoattractant protein-1 (MCP-1)	Chemokine involved in monocyte recruitment, contributing to inflammation and atherosclerosis in CAD.
Vascular endothelial growth factor (VEGF)	Associated with angiogenesis, may influence vascular health and contribute to CAD pathology.

Prior or Current therapy for autoimmune illness or cancer, diabetes, smoking, use of NSAIDs before blood collection, postmenopausal women, and anyone over the age of 65 were all excluded. There were no lipid-lowering drugs given to the participants. The blood is taken into EDTA collecting tubes and placed on ice immediately. Separate plasma from samples by centrifuging, then aliquot and store at −80°C until further use. Before detecting cytokine levels using the Thermosphere/Life Sciences 45-fold Human ELISA kit, samples must be thawed on ice per the manufacturer's instructions. Using a standard curve for each cytokine, the exponent program was used to analyze the raw data and convert them to pg./mL. Table 2 presents demographic and clinical data for the study cohort.

**Table 2:** Analyzing pooled plasma samples allowed researchers to compare the demographic traits of CHD patients with those of healthy controls.

Gender	CAD	Control	Total (values of *n* = 1,040)
Male	187	253	440
Female	203	397	600
Overall	390	650	1,040

### 3.2 Pre processing steps

The characteristics of the information are normalized before operating the classifier on the test to prevent predicted cues with greater values from overwhelming those with smaller values, which could lead to biased categorization. In addition, domain experts review and adjust the data for anomalies, such as nulls or outliers. The data is synthetically incremented by a smoothing bootstrap method because of considerably imbalanced tiny data sets, which minimizes overfitting in the training phase and translates to the testing phase, which is higher generalizability. Using the R programming language, ROSE Package enhances data synthesis to 1,000, balancing CAD in 52 percent of cases and Controls in 48 percent. By emulating a smooth bootstrap technique, the ROSE Package aids in these endeavors. Z-score normalization is used to scale the data. The resulting balanced data, which contains 52 percent (“CAD”) and 48% percent Control, is suitable for LSTM and CNN implementation. When data is expanded to one thousand observations and combined with balance data, standard partitioning for training and testing partitions can be used 75-25 percent of the time. This split will enable observations to be included in the training dataset, preventing model underfitting.

**Figure 1:**
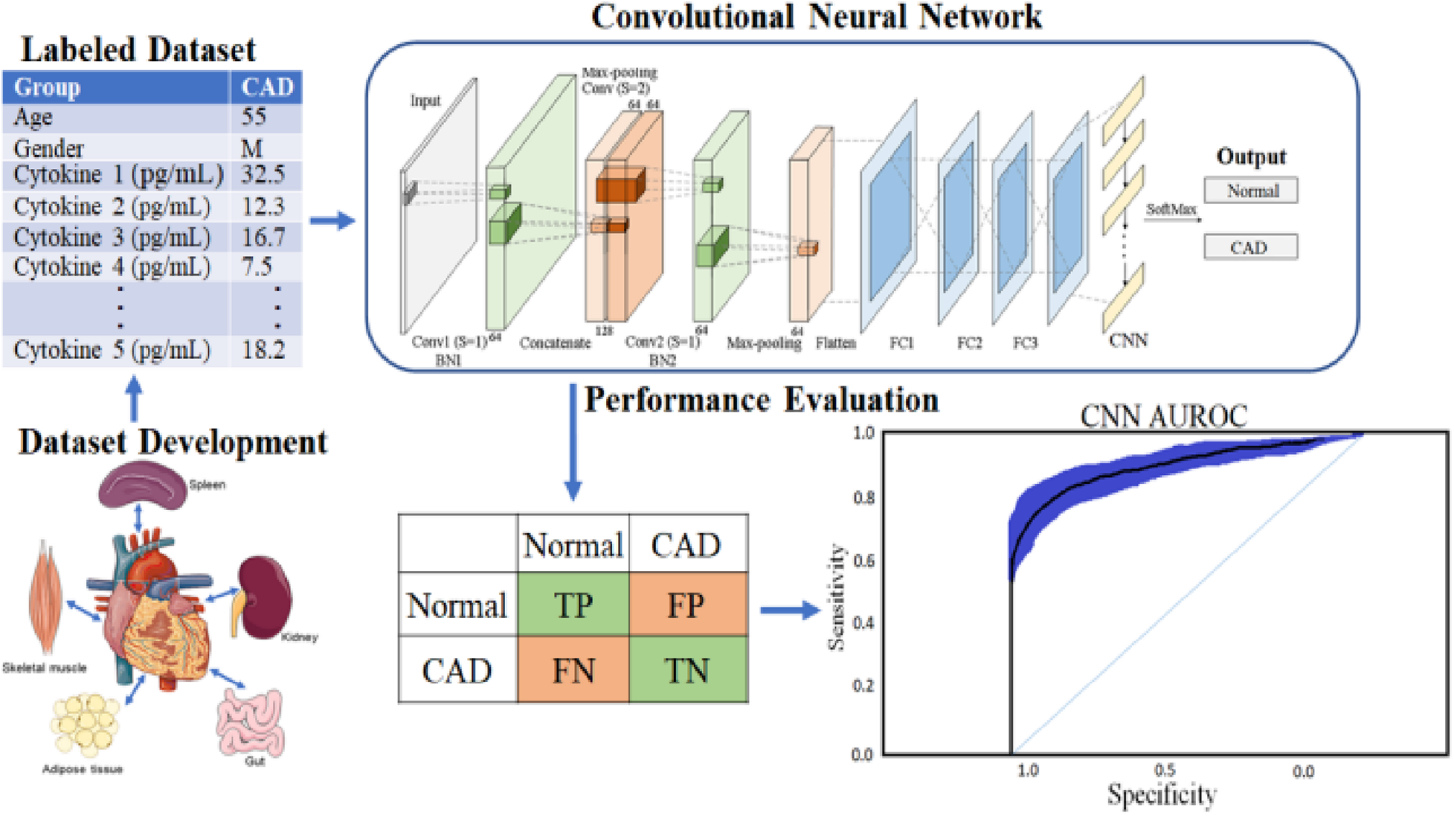
Proposed deep learning framework for CORONARY ARTERY DISEASE detection.

In addition, to avoid biased predictions, a 10-fold cross-validation with three replications was used to choose hyperparameters. Because feature selection was unnecessary after balancing and adding data to avoid model underfitting or overfitting, a total prediction feature space of 450 cytokines was used. The proposed CAD framework is depicted in Figure 1. The optimal balancing of bias-variance (underfitting and overfitting) tradeoffs is achieved using these strategies.

### 3.3 Convolutional neural network

A convolutional neural network (CNN or ConvNet) is a framework that leverages patterns in data, such as photos, videos, text, and bioinformatics sequences, to automatically recognize and classify information (35). CNNs are particularly useful for evaluating time series and signal data, and the structure and function of the brain's visual cortex strongly influence their design. The visual cortex comprises stratified tissue and two fundamental cell types: simple and complicated. Simple cells respond to primitive patterns in the subregion of visual stimuli, while complex cells use this information to identify more intricate characteristics.

**Figure 2:**
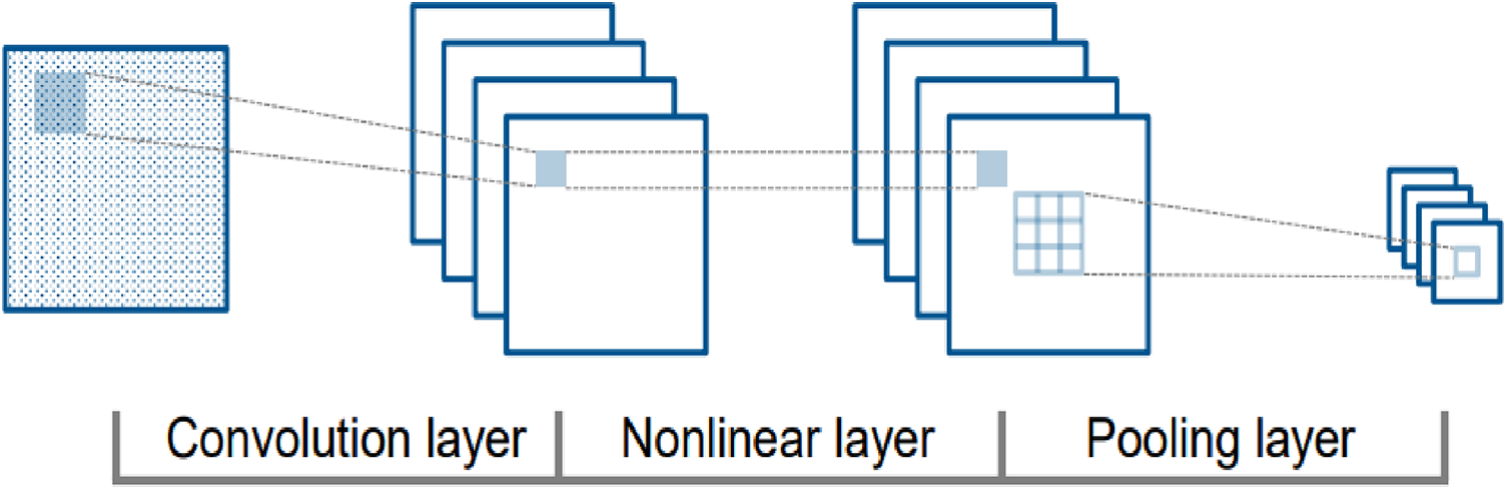
CNN structure with convolution, nonlinear, and pooling layers.

CNNs duplicate the visual cortex by utilizing three essential concepts: local connection, localization invariance, and local transition invariance. As shown in Figure 2, the fundamental structure of a CNN involves different nonlinear layers (36). Filters or weight vectors are applied to local data blocks to generate feature maps at each convolutional layer. These feature maps are then utilized to generate feature maps at a higher level. The recurrent application of filters throughout the dataset increases training efficiency by decreasing the number of parameters to be learned. The nonlinear layers are then blended to enhance the map's nonlinear characteristics. Subsampling non-overlapping regions with maximum or average values, the clustering layer further aggregates local characteristics to uncover more complex features (37).

### 3.4 Deep network

**Figure 3:**
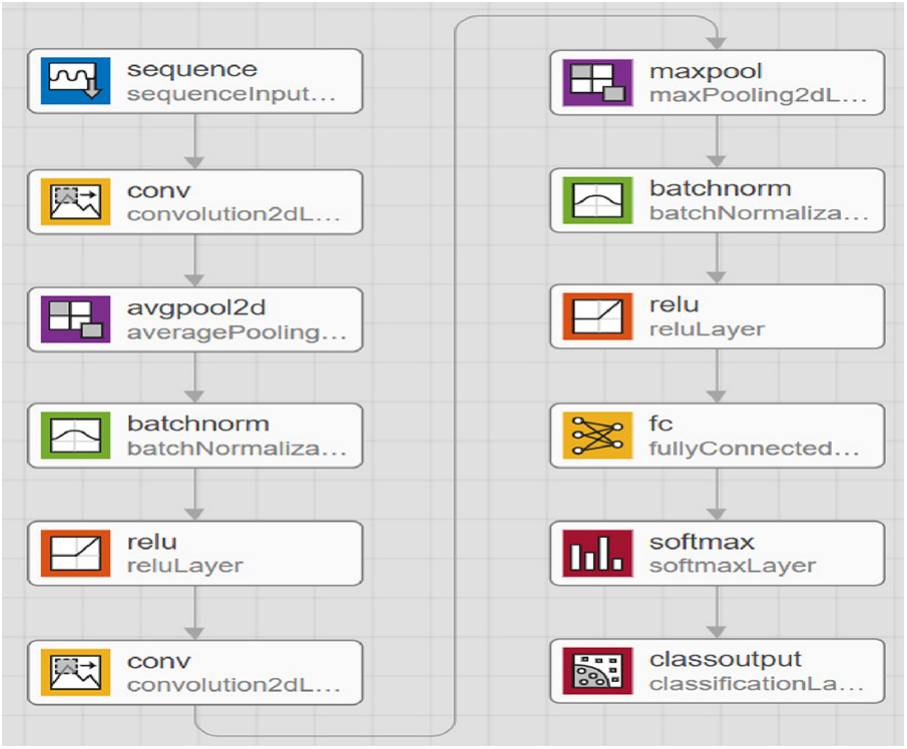
Proposed convolutional neural network model.

A deep network that uses data operations to learn specific features from the data. As depicted in Figure 3, a CNN consists of multiple layers, including a convolutional, activation, and pooling layer. The convolutional layers pass the input image through several convolutional filters, each activating a distinct image feature. This method, known as feature extraction, finds patterns in your data. The activation layer is often implemented as a rectified linear unit (ReLU) for faster and more efficient training. This method is sometimes called feature selection since only enabled features are passed to the subsequent layer (38). Table 3 presents the detailed architecture of the proposed CNN, providing information on the input and output of each layer, as well as the network training parameters.

**Table 3:** CNN architecture and parameters of the proposed deep learning model.

Layer type	Input/output	Shape	Parameters
Input sequence	Sequence	(Seq, Features)	0
Conv2D	Feature vector	(1, 1, 32)	448
AvgPooling2D	Feature vector	(1, 1, 32)	0
Batch normalization	Feature vector	(1, 1, 32)	64
ReLU	Feature vector	(1, 1, 32)	0
Conv2D	Feature vector	(1, 1, 64)	4,640
MaxPooling2D	Feature vector	(1, 1, 64)	0
Batch normalization	Feature vector	(1, 1, 64)	128
ReLU	Feature vector	(1, 1, 64)	0
Flatten	Feature vector	(64)	0
Fully connected	Feature vector	(2)	2,171,392
Softmax	Probabilities	(2)	258
Classification output	Probabilities	(2)	0
Total parameters			2,178,130

The clustering layer loses the number of considerations the system needs to discover by subsampling the output nonlinearly. This minimizes computing complexity and increases the network's resiliency. CNN architecture moves on to classification after learning multi-layer features. The next layer is the fully connected layer, which outputs a K-dimensional vector, where K is the number of predicted classes. This vector includes the likelihood that each class in an image is classified. The classification layer, the final layer of the CNN design, uses methods such as softmax to produce the result. This process is performed multiple times, and each layer learns to distinguish unique features. CNNs can learn and detect increasingly complicated data patterns by stacking numerous layers.

### 3.5 Recurrent network

A recurrent network like RNN was created mainly for sequential data handling. Circular connections within hidden cells enable circular computing. This circular computation implicitly enables the network to store past data as a state vector. This information is utilized to calculate the output of a specific input in addition to the current input. Long-term memory networks (LSTMs) are a popular type of RNNs commonly employed in applications such as speech recognition, where previous and future inputs influence outputs. LSTM is designed to overcome the leaking gradient problem, a prevalent issue in RNNs that makes learning long-term dependencies challenging. Table 4 showcases the comprehensive architecture details of the proposed RNN-LSTM model. It elucidates the input and output specifications of each layer while also offering insights into the network's training parameters.

**Table 4:** RNN-LSTM architecture and parameters of the proposed deep learning model.

Layer type	Input/output	Shape	Parameters
Sequence input	Sequence	(Seq, Features)	0
LSTM	Sequence	(Seq, 128)	98,816
LSTM	Feature vector	(64)	49,408
Reshape	Feature vector	(1, 1, 64)	0
Conv2D	Feature vector	(1, 1, 32)	18,464
AvgPooling2D	Feature vector	(1, 1, 32)	0
Batch norm	Feature vector	(1, 1, 32)	128
RELU	Feature vector	(1, 1, 32)	0
Conv2D	Feature vector	(1, 1, 64)	36,928
MaxPooling2D	Feature vector	(1, 1, 64)	0
Batch norm	Feature vector	(1, 1, 64)	256
RELU	Feature vector	(1, 1, 64)	0
Flatten	Feature vector	(64)	0
Fully connected	Feature vector	(2)	130
Softmax	Probabilities	(2)	0
Total parameters			184,130

The RNNs can develop deeper structures over time and make systems appropriate for sequential data applications such as natural language processing, language translation, and time series prediction. In recent years, RNNs have been successfully employed in multiple domains thanks to the development of increasingly complicated hidden units, such as LSTM memory cells, drastically reducing the gradient vanishing issue. RNNs have additional prospects for more advanced applications, including speech recognition, synthesis, and image captioning.

**Figure 4:**
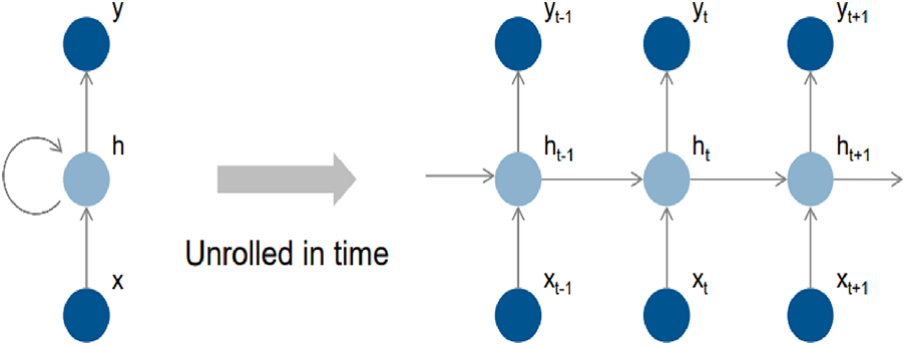
Recurrent features learning block of LSTM classifier.

Figure 4 depicts the fundamental structure of a recurrent neural network (RNN), which consists of input cells (x), hidden cells (h), and output cells (o) (y). The hidden cells are designed to use circular joins to handle sequential information, allowing computation to consider inputs from hidden cells from earlier time steps and current input cells. This circular join enables the network to store past information implicitly in hidden units called state vectors and to use state vectors to compute the output of the current input while accounting for all previous inputs. If the RNN grows over time, it can reflect circular computation more precisely. Each time step is represented by an index in this representation, and the masked unit (h) gets input from the input unit (x) and the masked unit from the previous time step (h-1). The calculation result is then transmitted to the output (y) and the masked cell for the subsequent time step (h + 1).

**Figure 5:**
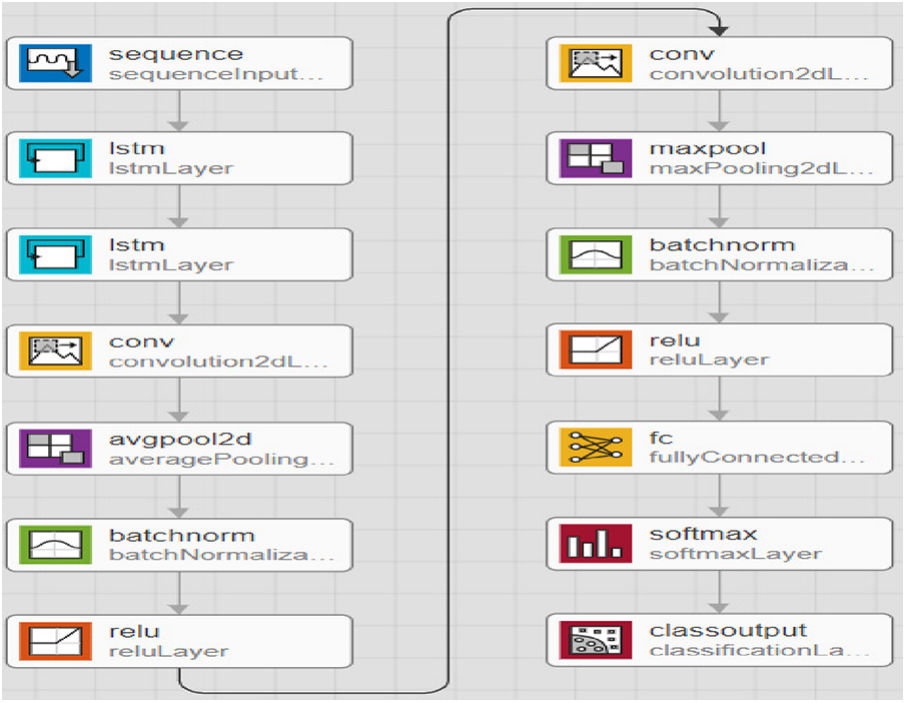
Proposed extended short-term memory-based recurrent neural network model.

In Figure 5, a feature learning block with an LSTM node is added to the neural network. The input features are first converted to sequential data, where each data point is linked with the next and previous data points to add up some sequential information by matching the same patterns in data points. Instead of a single LSTM layer, multiple LSTM is used to create a sequence-to-sequence model, where the input to the first LSTM is a sequence, and the output of the first LSTM is also a sequence. Similarly, the second LSTM accepts sequence as input but generates a features map as output. The feature map passes through several convolutional operations for learning robust and optimal features. The last two layers of the LSTM-RNN are SoftMax and classification, which are responsible for probability calculation and prediction, i.e., assigning a class label to the test sample.

### 3.6 K-fold cross validation

To improve the final classification results, combine k-fold cross-validation with an optimization method considering statistically significant cytokines. The k-fold cross-validation methodology improves the prediction power of fresh unlabeled data while avoiding problems like overfitting and variety bias. The method investigated by a (10-fold) cross-validation with (k = 10) was performed three times.

The CNN technique, which involves 450 cytokine prediction characteristics with Euclidean distance, was used in the first classifier experiment. The second classifier used 450 cytokines to create an RNN-LSTM. The experimental framework classifier is depicted graphically in the following image, Figure 1.

A general set of performance evaluation measures can be applied to acquire insights into the algorithm's correctness. In this study, we used AUROC to compare the performance of the developed classifiers and to estimate the precision & discriminating the CAD group from the control group.

### 3.7 Area under receiver operating characteristic (AUROC)

This is a commonly used statistic for determining the degree of separability across different classification algorithm implementations. A higher AUROC indicates that the algorithm can accurately classify examples into objective sets. An AUROC graph is generated by mapping false-positive rates on the X-axis in opposition to true-positive rates on the Y-axis. AUROC provides an excellent technique to compare different classifiers’ statistical consistency and discrimination accuracy.

## 4 Experimental result

**Figure 6:**
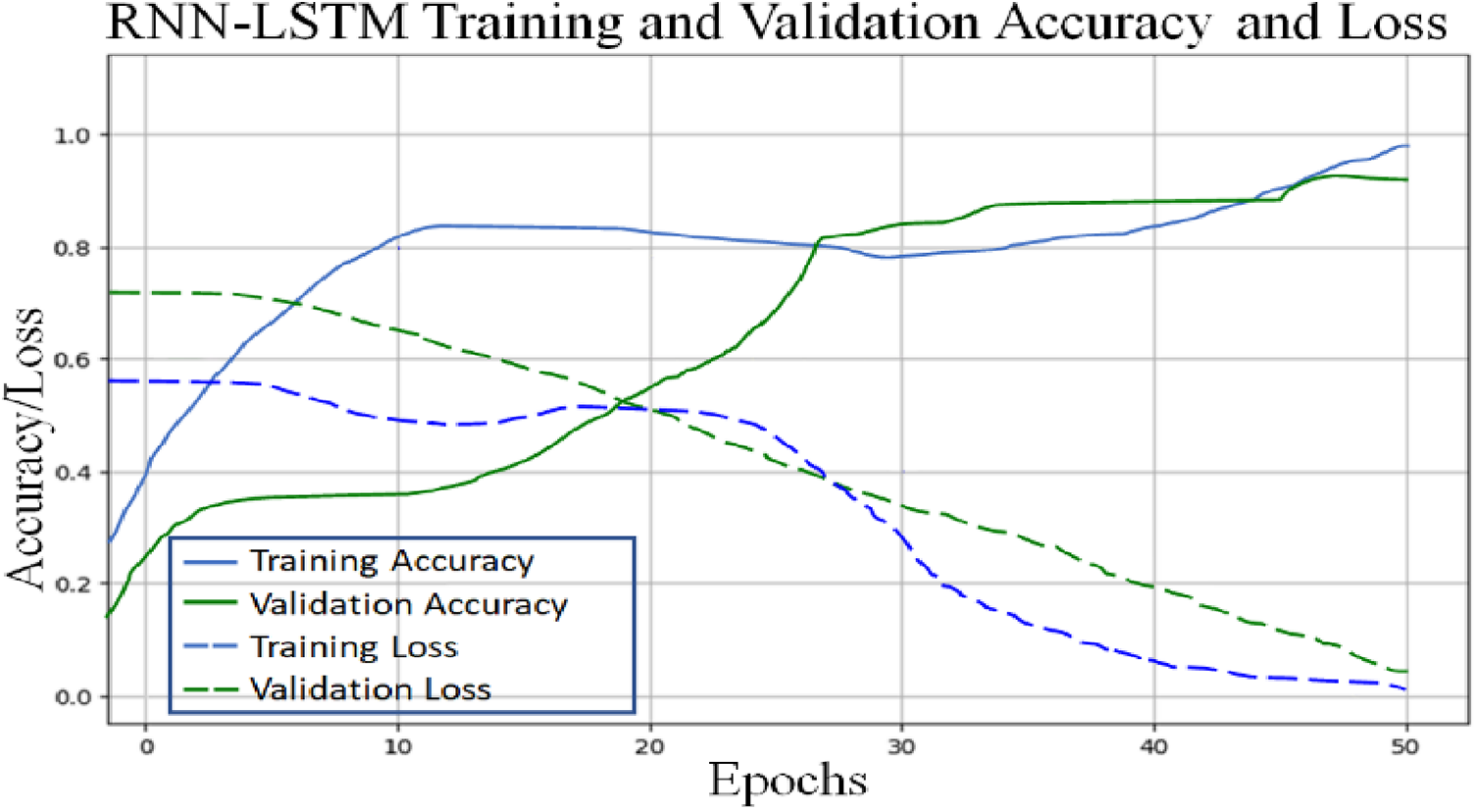
Proposed RNN-LSTM architecture validation accuracy and loss plot.

**Figure 7:**
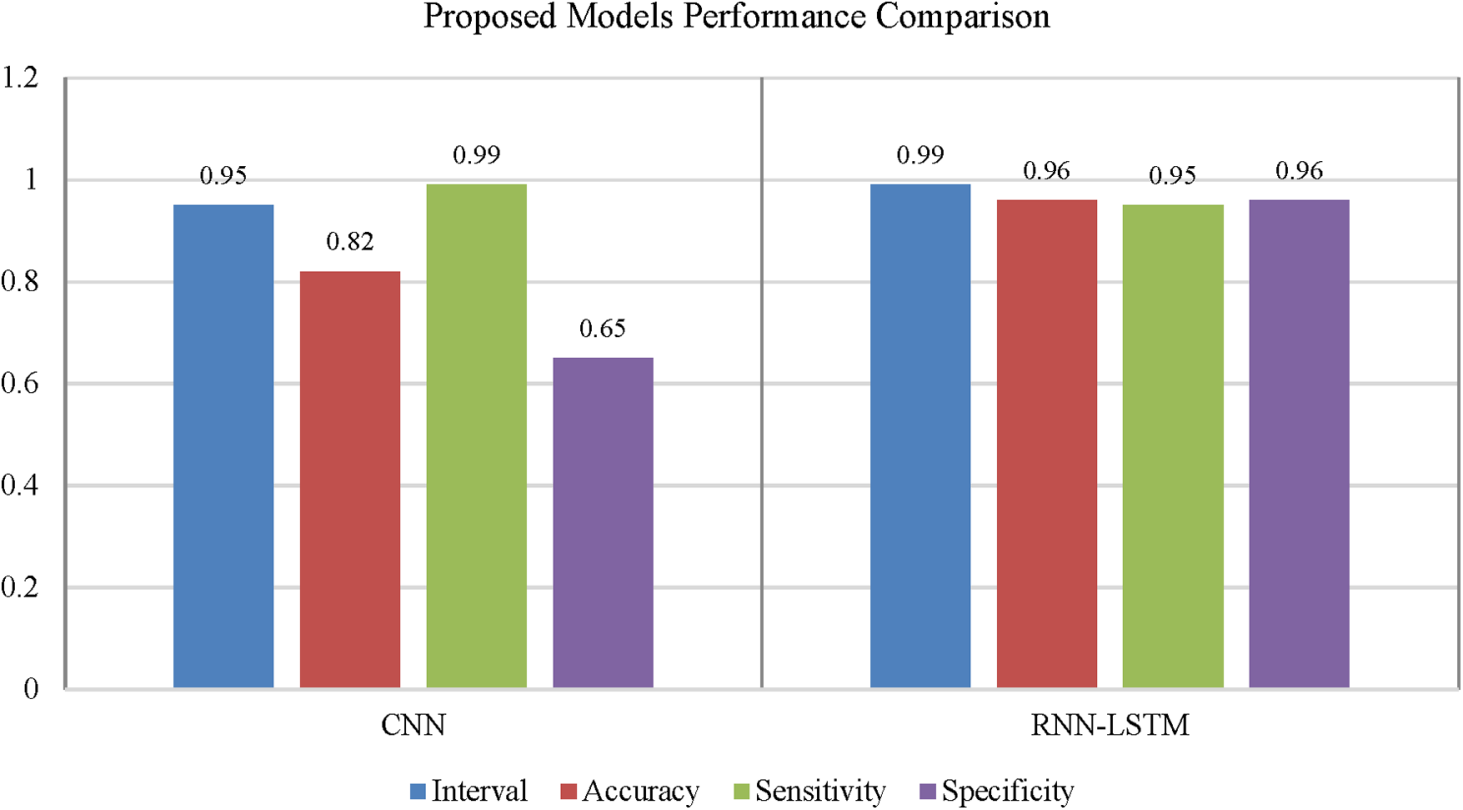
Performance comparison of convolutional neural network and recurrent neural network for coronary artery disease detection.

**Figure 8:**
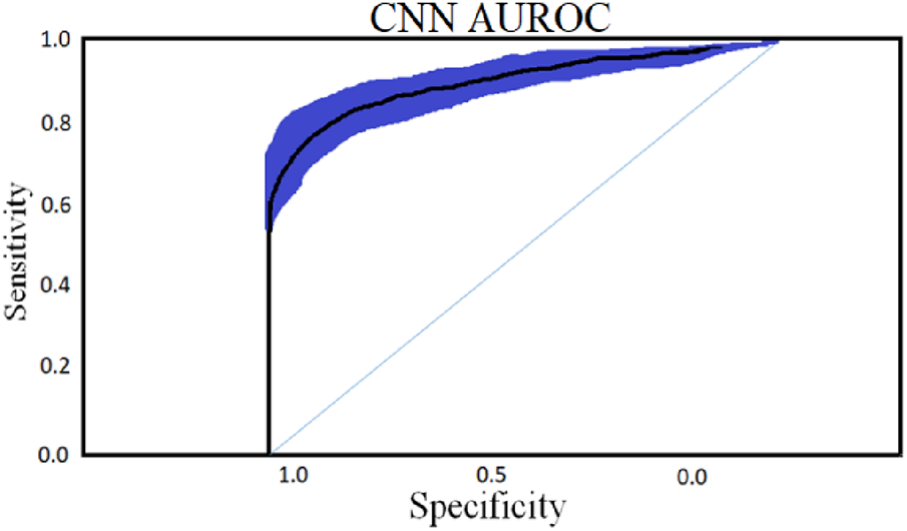
CNN classifier with an optimal epoch value 50 for 450 cytokines.

The accompanying tables (Table 5) and graphs demonstrate the test results produced by executing the algorithm on the test data (Figures 6–8). The hyperparameters tuning measure was adjusted by resampling replicas of the cross-validation results in the test results.

**Table 5:** Results comparison of proposed CNN and RNN (LSTM) classifier with 450 cytokines.

Algorithm	CNN	RNN-LSTM
Classification criterion		LSTM Stacking
	Optimizer = “Adam”	Optimizer = “Adam”
	Epoch = 50	Epoch = 50
	Batch size = 8	Batch size = 8
Predictor feature space	450 cytokines	450 cytokines
AUROC with 95% CI	0.954 (.929,.979)	0.99 (.982,.999)
Prediction accuracy	0.832	0.96
Sensitivity	0.992	0.954
Specificity	0.658	0.967

### 4.1 Convolutional neural network

**Figure 9:**
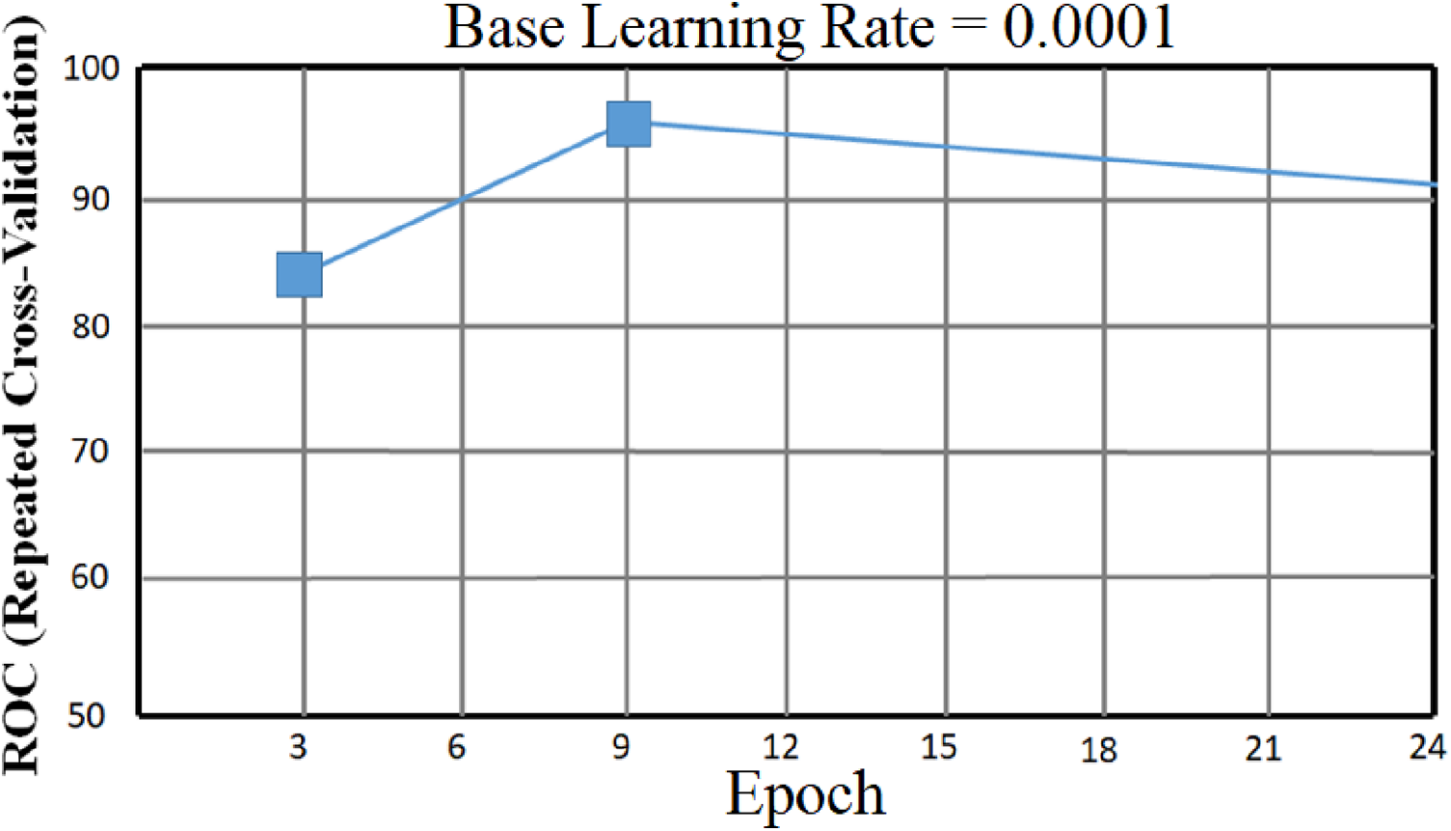
CNN classifier AUROC curve with 95% CI with an optimal epoch = 50 for 450 cytokines.

**Figure 10:**
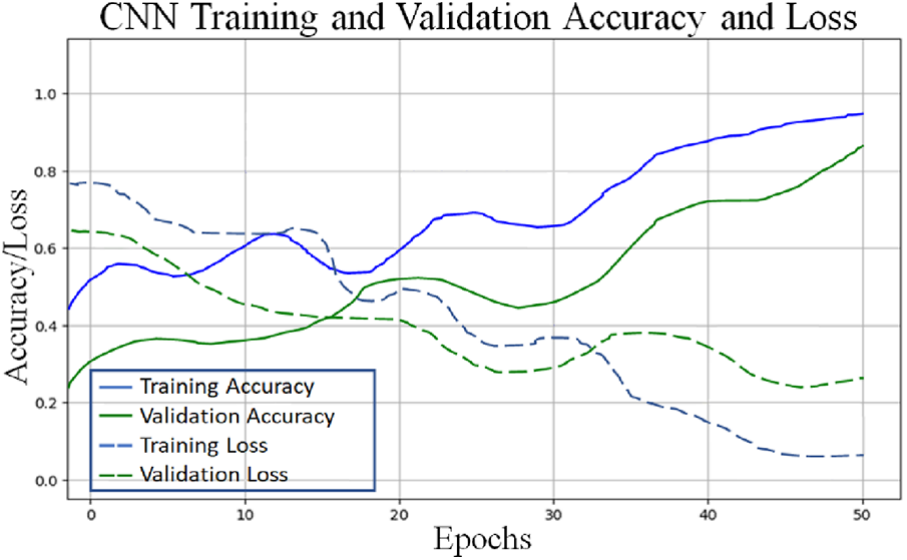
Proposed CNN architecture validation accuracy and loss plot.

To classify “at risk” instances, the classifier utilizes the CNN using the “Adam” optimization technique for optimal weights calculation. A total of 450 cytokines were employed in this classification. The AUROC value of 0.95, representing the degree of separation between CAD and the control, is quite essential. The above table and graphs provide detailed information on the AUROC, the optimal CNN neighbors determined by cross-validation, and the numerical metric of classifier 1. (Table 5 and Figures 8, 9). The Figure 10 illustrates the smooth training of the proposed CNN model. The model was trained for 50 epochs, exhibiting higher training and validation accuracy.

### 4.2 RNN-LSTM classifier result

**Figure 11:**
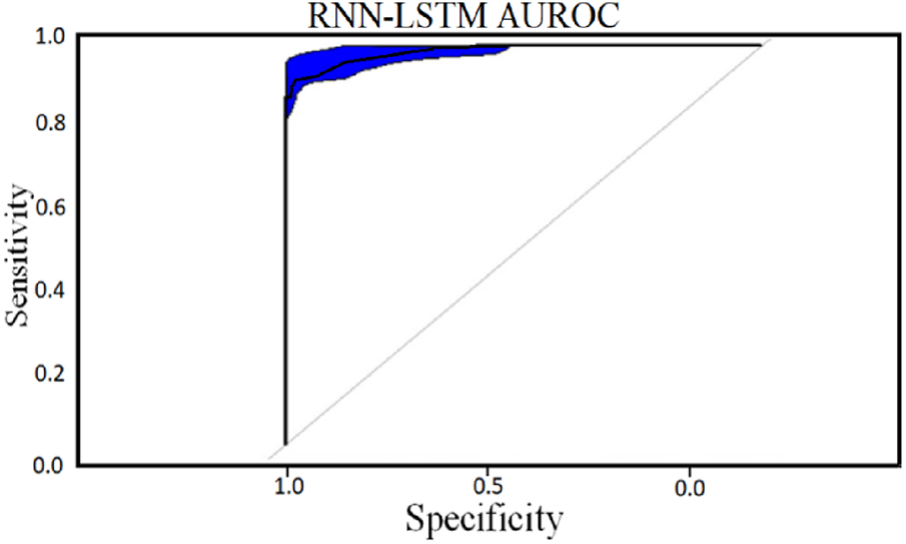
RNN-LSTM classifier AUROC curve for 450 cytokines.

Classifier 2 uses 450 cytokines as predictor characteristics in an RNN-LSTM. The AUROC of 0.99 is extremely high, and it outperforms previous classifiers in terms of accuracy. The foundation of the RNN-LSTM algorithm explains this achievement. For the RNN-LSTM classification, Stacked LSTM layers are used for learning the plasma cytokines data, six feature variables as segmentation standard, and the Gini index is a quality assessor were employed as hyperparameters. Combining random feature segmentation criteria, several LSTM layers and the accumulation of intermediate outcomes from these LSTM resulted in excellent results. Many LSTM units utilized the decision-making method to improve the final forecasts “accuracy” and “stability”. The graph and table below show the final numerical metric results and the AUROC for Classifier 2. (Table 5 and Figure 11).

### 4.3 Performance comparison of CNN and LSTM

**Figure 12:**
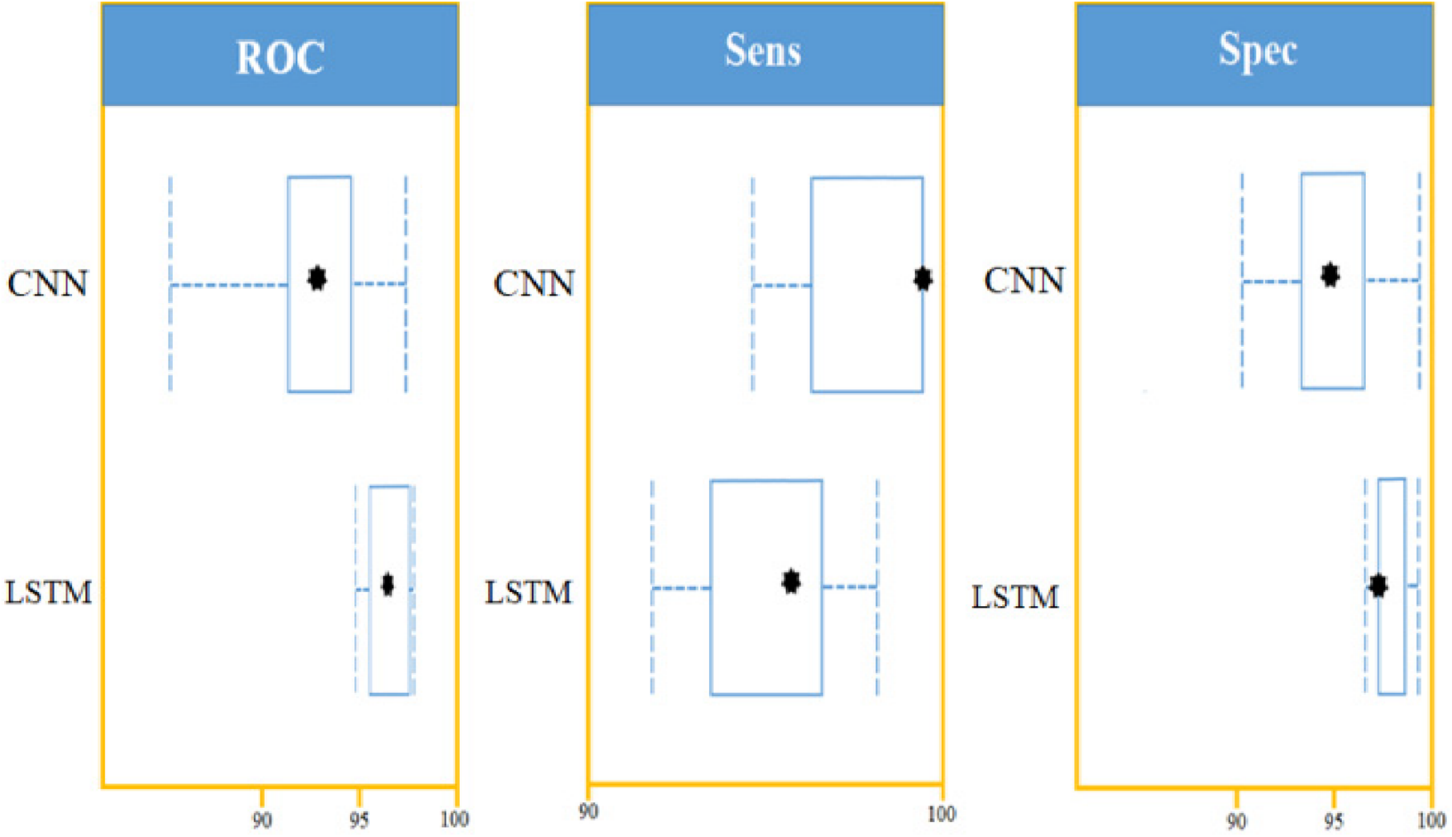
LSTM and CNN classification model comparison using: AUROC curve, sensitivity, and specificity on a set of 450 cytokines.

Using confidence intervals, the procedure can be combined with a *t*-test to check for significant differences in AUROC scores. A box-line plot can be used to compare the two classifiers visually. With *p*-values smaller than 7.48, independent *t*-test comparisons show two classifiers have significantly different AUROC values. The following figure shows paired box plot comparisons of AUROC, sensitivity, and specificity (Figure 12).

Table 6 provides a comparative analysis between the proposed method, which incorporates CNN and RNN-LSTM, and other state-of-the-art (SOTA) approaches in detecting coronary artery disease using plasma cytokine data. The results indicate that the proposed method outperforms the other models regarding prediction accuracy. Specifically, the CNN model achieved an accuracy of 83%, while the RNN-LSTM model demonstrated an impressive accuracy of 96%. These accuracies surpass the performance of several well-known models commonly found in the literature, such as K-Nearest Neighbor, Random Forest, Logistic Regression, Artificial Neural Network, AdaBoost, SVM, KNN, Bagging, Gaussian Naive Bayes, Decision Tree, Naive Bayes, Random Forest, Support Vector Machine, and Extreme Gradient Boosting. These findings strongly suggest that the proposed method holds great promise as a practical approach for detecting coronary artery disease using plasma cytokine data.

**Table 6:** Performance comparison of proposed deep learning model with some SOTA approaches.

Article	Method	Accuracy
(39)	K-nearest neighbor	69.71 83%
	Random forest	81.33 96%
(40)	Logistic regression	62%
	Artificial neural network	74%
(41)	AdaBoost	79%
	SVM	82%
	KNN	75%
	Bagging	79%
	Logistic regression	81%
	Gaussian naïve bayes	80%
(42)	Decision tree	82
	K-nearest neighbor	79
	Logistic regression	65
	Naïve bayes	79
	Random forest	64
	Support vector machine	74
	Extreme gradient boosting	69
Proposed	CNN	83%
	RNN (LSTM)	96%

## 5 Discussion

With the experiment of RNN-LSTM classifier, 2 (450 cytokines) was shown that the ML is a reasonable classification technique overall, with a score of AUROC (99%) and a (95%) confidence interval of “95” percent (“.982,.999”). Figure 7 compares the two proposed deep-learning models for coronary artery disease classification concerning performance. The data comprises a collection of non-sequential data that work together to produce better classification than the CNN model; RNN-LSTM outperforms other models and achieves almost perfect AUROC. RNN-LSTM, like stacking, uses bootstrap samples to form a deep network. RNN-LSTM also picks a subset of sequences for each partition they construct. This desirable trait leads to single output predictions with more diversity and uncorrelated prediction errors. Creating the classifier with a random subset of characteristics at each partition point allows for more diversified integration and, thus, higher overall performance than methods like CNN.

The (AUROC) value of (0.955) with 95% percent CI for the (CNN) with “450” cytokines (Classifier 1 experiment) (“.929” “.979”) is an excellent but not better than Classifier 2, which gave near good control differentiation and CAD. The RNN-LSTM classifier (classifier 2) considerably outperformed the CNN classifier (*p*-value 7.48) in a *t*-test comparing the AUROC scores of the two classifiers (*p*-value 7.48) (classifier 1).

Due to the incredible processing power and excellent predicted accuracy, ML algorithms are becoming more widely used, which is an improvement over current qualitative picture analysis and basic quantitative assessments of heart anatomy and function. ML algorithms can build a holistic framework incorporating images and other valuable aspects for trustworthy insight and early diagnosis that can save lives.

The reviewed studies and current research emphasize the status of the machine learning approach and how it can be utilized to detect patients at higher risk for (CAD) and guide conventional treatment options. RNN-LSTM is a deep-learning classification algorithm popular and used in various domain areas, including medical diagnosis. At the same time, CNN is an algorithm of similarity metric based. RNN-LSTM used in this research is stacked LSTM, where the output of the first LSTM is also a sequence that is forwarded to the next LSTM layer for learning purposes; LSTM is very popular for learning sequential data and is used in a wide range of domain areas; including medical diagnosis. Some of the most well-known are discussed here. The current analysis shows outstanding prediction accuracy, a significant improvement over Alizadeh Sani et al. research work (13). The RNN-LSTM highest AUROC used in this investigation received (.99), outperforming Yu et al. review study (15). Traditional procedures, such as angiography, cannot be replaced by this prediction system. It can, however, suggest more advanced tests for people at risk of developing a more severe disease. When used and compared, multiple methods are an effective way to gain a categorized overall perspective. This comparison paradigm is combined in the current investigation to present (23) results. The current study's in-depth investigation was guided by empirical evidence of performance enhancement using hyperparameter change and (cross-validation) (43) with RNN-LSTM and CNN. In general, fine-tuning hyperparameters has proven to be a successful optimization strategy. (Novel, cytokine, biomarkers) as indicators of inflammation (9, 44, 45) could be employed to predict CAD risk and serve as a comprehensive therapy target in the future more accurately.

## 6 Conclusion

This study is the first to employ cytokine plasma indicators to distinguish CAD from non-CAD sufferers. It also emphasizes the experimental methodology of multiple classifier studies, which demonstrate better-predicting accuracy across models. The performance of the CNN algorithm implementation is compared to that of the RNN-LSTM method in terms of efficacy. In comparison to earlier research employed RNN-LSTM and (“cytokines”) to identify and control disease groups, this study had a higher AUROC (0.99). For all 450 cytokines, mutually forest and “k—NN” produced reasonable effects. Cross-validation, data balancing, data augmentation, and employing 75-25 percent of the training and test sets splits were used in the CNN and RNN-LSTM classifier trials to balance the bias-covariance tradeoff. Overall, the RNN-LSTM has an AUROC of 0.99 with a 95 percent confidence interval of (0.982,.999), a prediction accuracy of 0.96, and a *p*-value is less than 7.480e-10. Utilizing the optimization, generalization, computational, and abstraction power of universal ML is vital and is employed in a wide range of fields in this era of creative and universal ML systems. Medicine is a well-known profession. Future research on the role of cytokine profiles in detecting the inflammation that CAD patients experience will lead to treatment targets. Many biological variables, including species of molecular lipoproteins, genetic drivers, oxidative stress of coagulopathy, and inflammation, have been demonstrated to contribute to CAD risk in recent studies. The analytical mathematical approaches developed in this study will allow for investigating various elements in estimating CAD risk, including their interactions. Adding a wide range of cytokines will provide additional aspects to this study, allowing for better risk prediction and new treatment options.

## Data Availability

The datasets presented in this study can be found in online repositories. The names of the repository/repositories and accession number(s) can be found below: https://figshare.com/, https://figshare.com/articles/dataset/Coronary_Artery_Disease_Plasma_Cytokine_Data/24942093.
